# The Enzyme Effect: Broadening the Horizon of MS Optimization to Nontryptic Digestion in Proteomics

**DOI:** 10.1021/jasms.4c00396

**Published:** 2025-01-13

**Authors:** Kinga Nagy, Péter Sándor, Károly Vékey, László Drahos, Ágnes Révész

**Affiliations:** † MS Proteomics Research Group, HUN-REN Research Centre for Natural Sciences, Magyar Tudósok körútja 2, H-1117 Budapest, Hungary; ‡ Hevesy György PhD School of Chemistry, ELTE Eötvös Loránd University, Faculty of Science, Institute of Chemistry, Pázmány Péter sétány 1/A, Budapest H-1117, Hungary

**Keywords:** enzyme effect, MS optimization, nontryptic digestion, proteomics

## Abstract

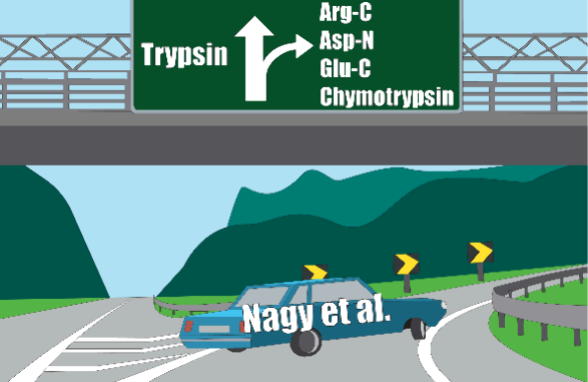

In recent years, alternative enzymes with varied specificities have gained importance in MS-based bottom-up proteomics, offering orthogonal information about biological samples and advantages in certain applications. However, most mass spectrometric workflows are optimized for tryptic digests. This raises the questions of whether enzyme specificity impacts mass spectrometry and if current methods for nontryptic digests are suboptimal. The success of peptide and protein identifications relies on the information content of MS/MS spectra, influenced by collision energy in collision-induced dissociation. We investigated this by conducting LC-MS/MS measurements with different enzymes, including trypsin, Arg-C, Glu-C, Asp-N, and chymotrypsin, at varying collision energies. We analyzed peptide scores for thousands of peptides and determined optimal collision energy (CE) values. Our results showed a linear *m*/*z* dependence for all enzymes, with Glu-C, Asp-N, and chymotrypsin requiring significantly lower energies than trypsin and Arg-C. We proposed a tailored CE selection method for these alternative enzymes, applying ca. 20% lower energy compared to tryptic peptides. This would result in a 10–15 eV decrease on a Bruker QTof instrument and a 5–6 NCE% (normalized collision energy) difference on an Orbitrap. The optimized method improved bottom-up proteomics performance by 8–32%, as measured by peptide identification and sequence coverage. The different trends in fragmentation behavior were linked to the effects of C-terminal basic amino acids for Arg-C and trypsin, stabilizing y fragment ions. This optimized method boosts the performance and provides insight into the impact of enzyme specificity. Data sets are available in the MassIVE repository (MSV000095066).

## Introduction

Liquid chromatography–mass spectrometry (LC-MS) has become a powerful analytical tool in the protein search, including biomarker discovery and routine measurements for clinical diagnostics.^1−3^ A pivotal step in the bottom-up approach is enzymatic digestion, wherein proteins are cleaved into smaller peptides for subsequent identification and analysis.^4^ Trypsin has long been the “gold standard” enzyme for this purpose due to its specificity, efficiency, affordability, and good mass spectrometric properties.^4,5^ However, trypsin specifically cleaves at the C-terminal of basic amino acids lysine (Lys) and arginine (Arg), which results in one-third of the tryptic peptides consisting of less than 6 amino acids.^6,7^ The too short peptides (frequent from Lys/Arg rich histones) or too large peptides (typical from transmembrane proteins and due to missed cleavages) cannot be identified and sequenced confidently.^7−10^ Finally, neighboring negatively charged side chains, phospho-modified residues, and glycosylation may cause missed cleavages.^5,7,11,12^

These limitations led to the utilization of alternative enzymes either alone or in combination with trypsin.^13,14^ Their various different cleavage sites provide orthogonal information compared to trypsin and enables us a more comprehensive view of the proteome.^7,15−17^ Application of alternative enzymes can lead to improved protein sequence coverage even in the cases when only modest gain in the number of identified proteins is obtained.^9,10,18,19^ Furthermore, the production of overlapping peptides from multiple protease digestions is needed for de novo sequencing, reaching full sequence coverage, mapping protein isoforms, and detecting spliced junction sequences.^7,10,15,16,20^ There is also an opportunity to move in the direction of middle-down proteomics because peptides produced by alternative enzymatic digestions tend to be longer. These peptides can be separated more effectively, and their sequences are more likely to be unique.^6,21^ Alternative enzymes also enable the investigation of PTMs that cause problems when using trypsin for digestion.^5,8,9,18,22^ Furthermore, their use can expand digestion parameters,^8,9,23^ and the multienzyme approach may contribute to nonbiased quantitative results and various specific proteomic applications.^8,15,24^

Some of the most frequently used alternative enzymes are Lys-C, chymotrypsin, Glu-C, Arg-C, and Asp-N in proteomics. Lys-C is usually used for predigestion during tryptic digestion and cleaves at the C-terminal of Lys. Glu-C cleaves at the C-terminus of glutamic acid (Glu) and aspartic acid (Asp), while Asp-N cleaves at the N-terminus of the Asp. Chymotrypsin is a less specific enzyme; it cleaves at the C-terminus of several amino acids (alanine, Asp, Glu, leucine, methionine, phenylalanine, tyrosine, and tryptophan), which is why shorter peptides are produced during digestion. Like trypsin, Arg-C also cleaves at a basic amino acid at the C-terminus of Arg. The expected number of missed cleavages depends on the enzyme. During the database search, 0–2 missed cleavages are most often expected, but up to 4 missed cleavages can be set when using relaxed specificity settings.^25^

One of the most widely used fragmentation techniques in tandem mass spectrometry is collision-induced dissociation (CID), during which b- and y-ions are formed as major product ion types.^4,26^ These fragment ions provide structural information about the fragmented precursors, thus enabling the identification of peptides and, based on them, proteins. In the case of y-ions, the charge is retained on the C-terminus, while b-ions have charge on the N-terminus. In MS, fragmentation of peptides is largely driven by charge localization and the presence of mobile protons: fragment ions containing basic residues generally produce more intense peaks than fragment ions lacking basic residues.^23,27−30^ In line with this, in the case of tryptic peptides, y-ions–stabilized by basic amino acids Lys or Arg–are more stable and more abundant than b-ions, and their relative abundances are significantly different.^31−34^ However, this statement may not be true for peptides produced by alternative enzymes. The lack of a basic amino acid at the C-terminus may cause no robust y-ions compared to b-ions; rather, the position of the basic residue determines the intensity relationships.^10,23,35^

The most important MS/MS parameter in CID is collision energy (CE): the peptides fragment to different extents at different CE settings, and the relative ratio of the different fragment ions also varies with CE.^36^ The settings recommended by the manufacturer are not necessarily optimal, it may be worth fine-tuning the measurement parameters according to the goals of the project and to the actual LC-MS/MS platform used.^37^

The energy dependence of the fragmentation of tryptic peptides and the optimal CE choice from various proteomics point of view have been extensively studied in the past decades.^37−40^ However, when alternative enzymes are used, experimental parameters optimized for tryptic peptides are typically applied in the literature.^15,17,18,21,22,24,25,29^ For LC-MS/MS analysis of nontryptic peptides, only a handful of studies have investigated the effect of collision energy and only a couple of energy settings were investigated.^10,20,41^ It was demonstrated that the normalized collision energy (NCE%) generating the most intense MS/MS base peak (useful in determination of transitions during parallel reaction monitoring) is lower for all the investigated enzymes (Glu-C, Asp-N, Lys-C/Arg-C, and trypsin) than the setting typically used in proteomics; therefore, the difference between the enzymes was not found in this respect.^20^ Gadush et al. developed a *de novo* sequencing method for determining the complete protein sequence of monoclonal antibodies (mAbs) using a multienzyme approach involving trypsin, chymotrypsin, elastase, and pepsin and optimized the supplemental activation-NCE% values of EThcD.^41^ Again, no distinction between the enzymes was made. A recent prominent research work studied the comparison of trypsin, Glu-C, and Asp-N and investigated the efficiency of DDA analysis of HEK 293T lysate at NCE% of 24%, 27%, 30%, and 33%. Interestingly, the same settings (27 NCE% and 30 NCE%) were found optimal for all enzymes; although differences in the proportion of b- and y-type ions and their CE dependence were also obvious, their goal was to optimize a multiprotease DIA method for these enzymes.^10^

Even though the collision energy has a great influence on the MS/MS spectra, collision energy optimization in detail for digestions with alternative enzymes has not yet been carried out. Furthermore, analytical tools were also mainly optimized based on tryptic peptide properties which may bias the performance of other proteases in proteomics and results in lower scores for nontryptic peptide hits.^6,20^ In the present study, we aimed to optimize the MS/MS-based identification of nontryptic peptides focusing on four enzymes typically used in proteomics (chymotrypsin, Glu-C, Asp-N, and Arg-C) compared to trypsin as reference. We carried out several series of energy-dependent nano-LC-MS/MS measurements for various enzymatic digests to determine the optimal CE values for a large set of peptides. Both a standard sample (MS compatible human protein extract (intact, catalog no. V6941), which is a whole-cell protein extract prepared from human K562 cells and was purchased from Promega) and a complex biological sample (human blood plasma) were studied, and the data evaluation performance of two frequently used search engines, Byonic^42^ and Mascot,^43^ were compared. Differences were explained in light of different structural and fragmentation behaviors. Finally, based on the peptide level data, we set up an optimized workflow for nontryptic digests, which will lead researchers to maximize the performance of LC-MS/MS analysis for various frequently used enzymes.

## Experimental Section

### Chemical Reagents

Unless otherwise stated, reagents and consumables were from Sigma-Aldrich (Sigma-Aldrich Kft., Budapest, Hungary). MS compatible human protein extract (intact, catalog no. V6941), which is a whole-cell protein extract prepared from human K562 cells, was purchased from Promega (Madison, WI). Trypsin/Lys-C mix, trypsin, chymotrypsin, Glu-C, Asp-N, and Arg-C digestion enzymes were from Promega (Madison, WI). RapiGest SF was purchased from Waters (Milford, MA). Dithiothreitol and iodoacetamide for digestion and C18 spin column for cleanup were from Thermo Fisher Scientific (Waltham, MA).

### Digestion

Samples were subject to enzymatic digestion in aliquots of 15 and 25 μg in the case of human protein extract (intact) standard and human blood plasma, respectively. Tryptic digestion was carried out using our standard laboratory protocol: Briefly, denaturation of the proteins was performed by Rapigest SF, and the S–S bridges were reduced by dithiothreitol (30 min), followed by alkylation using iodoacetamide in the dark (30 min). Samples were digested first by Lys-C/trypsin mixture and then by trypsin. When using alternative enzymes, the samples were only digested by the given enzyme with a slightly different protocol based on a literature suggestion (see Table S1).^25^ Glu-C, chymotrypsin, Asp-N, and trypsin were applied in the case of human protein extract standard, and Glu-C, chymotrypsin, Asp-N, Arg-C, and trypsin were used in the case of human blood plasma. The appropriate pH was set using ammonium bicarbonate buffer solution. Digestion was quenched with the addition of formic acid. The digests were dried in SpeedVac, and cleanup was performed using C_18_ spin column (Thermo Fisher Scientific) using a protocol based on the manufacturer’s recommendation. After quenching and drying, samples were dissolved in injection solvent (98% water, 2% acetonitrile, and 0.1% formic acid) prior to the nano-LC-MS/MS analysis.

### Mass Spectrometric Analysis

Nano-LC-MS/MS studies of the digested human blood plasma and human protein extract samples were performed using our standard laboratory methods for proteomics investigations (see Material S1) with varying MS/MS collision energy settings. Briefly, in each run, 1 μg of human blood plasma (digested by Glu-C, chymotrypsin, Asp-N, Arg-C or trypsin) or 2–2.25 μg of human protein samples (digested by Glu-C, chymotrypsin, Asp-N, or trypsin) were subjected to nano-LC-MS/MS analysis using a Dionex Ultimate 3000 RSLCnano System coupled to a Bruker Maxis II ETD Q-TOF via a CaptiveSpray nanoBooster ionization source operated in positive mode. Peptides were separated on an Acquity M-Class BEH130 C18 analytical column using gradient elution following trapping on an Acclaim PepMap100 C18 trap column. Solvent A consisted of water + 0.1% formic acid, while Solvent B was acetonitrile + 0.1% formic acid. Spectra were collected using a fix cycle time of 2.5 s and the following scan speeds: MS spectra at 3 Hz over a mass range of 150–2200 *m*/*z*. CID using a default intensity threshold (15 000) was performed at 16 Hz for abundant precursors and at 4 Hz for peaks of low abundance. An active exclusion of 2 min was used after one spectrum, except if the intensity of the precursor was elevated 3-fold.

### Energy-Dependent Studies

**Figure 1:**
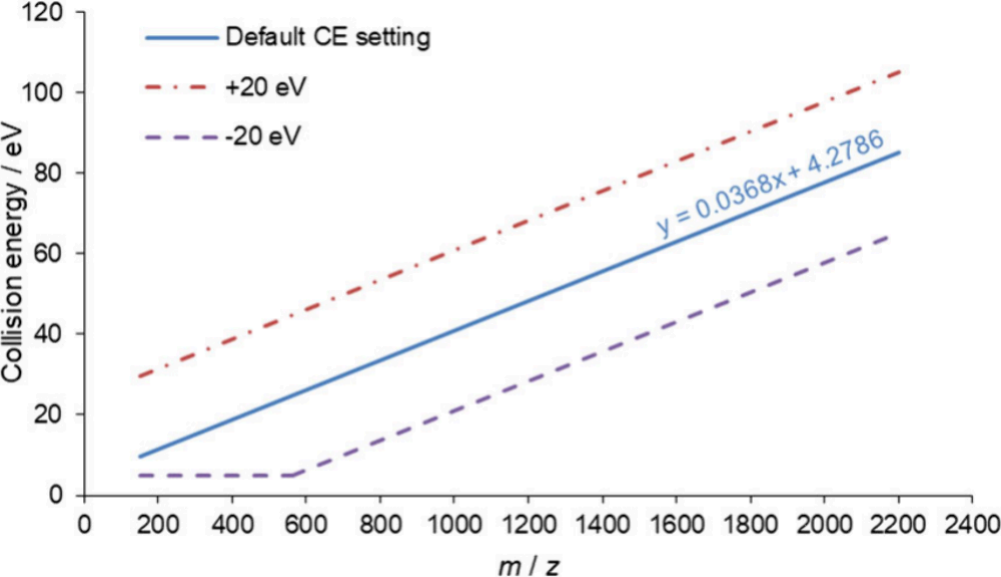
Investigated collision energy setting range in eV as a function of *m*/*z*. Blue line indicates the default collision energy setting (optimized for tryptic peptides), while dashed and dashed-dotted lines belong to the highest and lowest settings during the CE optimization process from −20 eV to +20 eV in 2 eV steps. The lower limit of the applied collision energy of the instrument was 5 eV; therefore, CE could not go below that value.

Our starting point was the collision energy setting previously determined and optimized for tryptic peptides of HeLa and *E. coli*.^34^ This CE setting includes collision energy values that are linearly dependent on precursor *m*/*z* considering the size of the species. We carried out further measurements at values higher and lower than this setting, from −20 eV to +20 eV, in 2 eV steps, resulting in 21 separate nanoLC-MS/MS measurements per enzymatic digestions of samples (see Figure 1). We highlight that the 2 eV steps in collision energy meant different step sizes in voltages for the different charge states.

### Data Analysis

The raw QTof data of measurements of human protein extract and blood plasma samples with different enzymatic digestions were first recalibrated using Bruker Compass DataAnalysis software v.4.3 (Bruker Daltonik GmbH, Bremen, Germany) for the internal calibrant. MS/MS spectra were searched against the fasta file from the human SwissProt database (August 2022) using Byonic and Mascot search engines. For alternative enzymes, the database search parameters are highly dependent on the digestion conditions.^25^ Regarding cleavage site specificity, missed cleavages, and also mass tolerance values and the list of variable modifications, recommendations of the Preview, a companion tool of Byonic, were used. The Preview recommended 2 missed cleavages for each enzymatic digestion; so, during each search, a maximum of two missed cleavages were allowed, and depending on the sample, trypsin, chymotrypsin, Glu-C, Asp-N, or Arg-C was set as enzyme (see Material S2 for further details).

### Determination of Optimal Collision Energies

For the determination of optimal collision energies of peptides, the evaluation of data was carried out analogously to our previous work.^34^ The Excel reports of Byonic and Mascot output files (.dat) were the input files for data aggregation carried out by the Serac program.^44^ For each peptide, the identification score was collected and plotted as a function of the collision energy. This was then normalized with the maximum score for the given peptide ion, and a Gaussian curve was fitted to the points, using the levmar^45^ and PGPLOT^46^ libraries. The positions of the center of the Gaussian peaks were considered as optimal collision energy values for the given peptides. Note that different charge states of the same sequence were treated separately.

To ensure confident peptide identifications, we only considered a peptide identified at a given collision energy if it met strict requirements. In the case of Mascot, a peptide was included in the energy-dependent analysis if its Mascot score exceeded 15 and if it was identified for at least 6 adjacent collision energies with a score above 25 at least at one collision energy.^34^ In the case of Byonic, the exclusion Byonic score was 100 and had to be identified for at least 6 adjacent collision energies with a score above 300 at least at one collision energy to ensure covering a high-quality peptide-spectrum match.^42^ The minimum peptide length was 6 with both search engines. Singly charged species were excluded from the analysis because they fragment less efficiently, thus providing spectra with less information, and often do not belong to peptide species. More details can be found in Supporting Information Material S3. The obtained peptide-level optimal CE values formed the basis of optimized setting determination (see Results and Discussion).

### Performance Test

Performance tests were performed with trypsin, Arg-C, Glu-C, and chymotrypsin digests of blood plasma examined at two collision energy settings optimized for tryptic peptides^34^ and nontryptic peptides (see Optimal Collision Energies of Nontryptic Peptides). During the experiments, all digests were measured with 3 repetitions at both MS settings, and data were evaluated using both Byonic and Mascot. For the performance test, we set the following filtering criteria: Byonic peptide identifications were accepted with a score >200 and log Prob value >2. In the case of Mascot, the Mascot ion score corresponding to *p* < 0.05 varied in the range from 20 to 29 for the different enzymes; on average, it was 25. Therefore, Mascot score >25 were required. The performance tests were characterized by the number of hits, score values, and sequence coverage. The peptide charges were merged with Serac, not considered separately.

## Results and Discussion

### Optimal Collision Energies of Nontryptic Peptides

The object of the paper was to optimize the MS-based identification of nontryptic peptides. In particular, we set out to investigate the collision energy dependence of peptides produced by alternative enzymatic digestions from the point of view of peptide identification, analogously to that of tryptic peptides.^34^ For this purpose, we conducted 5 series of nano-LC-MS/MS measurements on human blood plasma digested by Glu-C, chymotrypsin, Asp-N, Arg-C, or trypsin, as well as 4 series for the human protein extract digested by Glu-C, chymotrypsin, Asp-N, or trypsin. In total, 21 different collision energies were examined for all peptides, mapping the energy dependence in steps of 2 eV starting from the optimized setting for tryptic peptides.^34^

Overall, we could identify several hundreds of peptides from all nano-LC-MS/MS measurements using both Mascot (score >25) and Byonic (score >300) search engines from all investigated enzymatic digests. Among these, depending on the enzyme, 30–90% met the requirements set for energy-dependent analysis (see Determination of Optimal Collision Energies). The number of identified peptides are presented in the Supporting Information (Tables S2 and S3). Most of the peptides were doubly or triply charged, while the number of peptides with +4, +5, or +6 charge were too small for drawing reliable conclusions.

The CE dependence of identification scores was investigated by using both Mascot and Byonic search engines. Different charge states of the same peptide were treated separately. The identification score values of the peptides were collected as a function of CE setting, and CE-dependent curves were constructed for a large set of peptides with various enzymatic specificity. The obtained CE-dependent curves of the identification scores showed one or two maxima, which phenomenon was previously observed for tryptic peptides.^34^ When the CE-dependent curve of a peptide has one maximum, the peptide is called unimodal–so it has unimodal behavior; when a peptide has two maxima, it is called a bimodal peptide–so it has bimodal behavior (see a representative example in Figure S1). In the case of bimodal peptides, the two maxima are due to the different energy dependence of b and y fragment ion intensities.^34^ The positions of the center of the Gaussian peaks are considered as optimal collision energy values for the given peptides.

In the literature, studies on collision energy dependence and also MS instrument vendors generally reveal a linear trend between the peptide *m*/*z* and the optimal collision energy.^47−49^ This phenomenon is called degree-of-freedom effect and was investigated in detail for various type of species including peptides.^50^ In line with these results, manufacturers typically provide default CE methods linearly dependent on precursor *m*/*z* or this dependence is already hidden in the normalized collision energy (NCE) term (Thermo Orbitrap equipment).^49^ Our previous works with tryptic peptides already confirmed this linearity,^34^ and we got similar results for our current nontryptic peptides; although there is a large variance between the data points, this correlation is acceptable.

**Figure 2:**
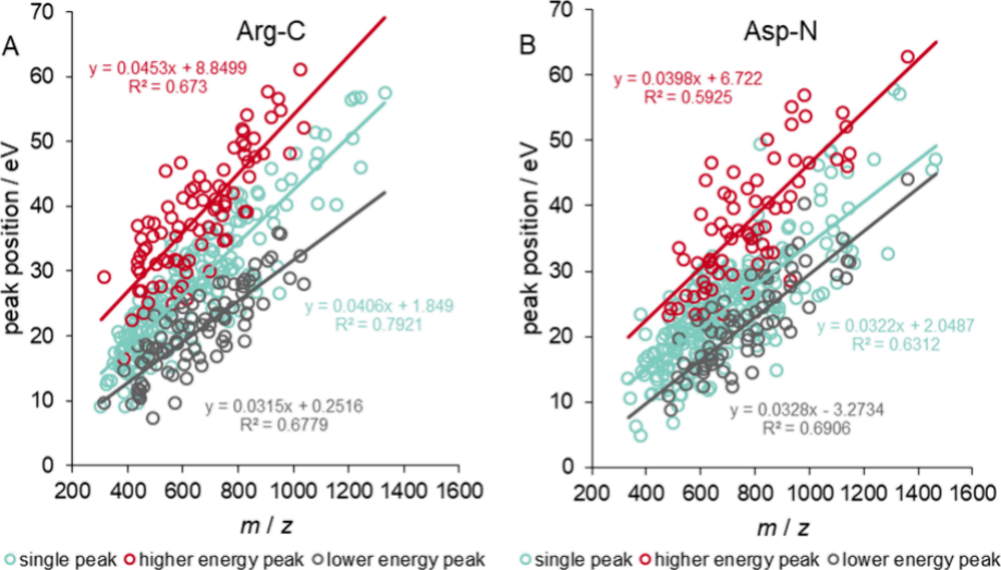
Peak positions (maxima of fitted Gaussian curve) in eV as a function of *m*/*z* for peptides with charge states from +2 to +6 from human blood plasma digested by (A) Arg-C and (B) Asp-N using the Mascot search engine. Light blue circles indicate the position of the sole peak for peptides having unimodal behavior, while red and gray circles are the higher and the lower optimal collision energies, respectively, for bimodal peptides. Straight lines represent linear fits. Note that, for the optimized CE settings, only the peak positions of the peptides with +2 and +3 were used.

The optimal CE values of the peptides were plotted as a function of their mass over charge ratio (*m*/*z*), including all charges (except +1) and peptides with +2 and +3 charges separately. Due to the unimodal and bimodal behavior of peptides, three different lines can be fitted into the three types of optimal CEs. In the case of tryptic peptides, we have already found that the line fitted to the unimodal optimum is approximately in the middle, between the two optimal CEs of the bimodal peptides.^34^ Furthermore, we previously proved that, in the case of tryptic peptides, it is worthwhile to set the device according to the line fitted to the unimodal CE values.^38^ We observed a very similar behavior for digestions with Arg-C (see Figure 2A), so we proceeded in an analogous way and accepted the line fitted to the unimodal optima as the optimal experimental setting. The similarity of Arg-C to trypsin can be traced back to the fragmentation properties of the peptides (see below), because both digestive enzymes cleave the protein chain at the C-terminus of basic amino acids (Arg vs Arg and Lys). For the same reason, although we did not examine it, we also expect this tendency for Lys-C, since it cleaves at the C-terminus of Lys as trypsin.

The trends for peptides produced by Asp-N, chymotrypsin, and Glu-C were very similar to each other but different from peptides produced by Arg-C and trypsin. As can be seen in Figure 2B in the case of Asp-N, the trendlines fitted to the unimodal optimum and the lower energy optimum of bimodal peptides are closer to each other than in the case of trypsin and Arg-C. Similar results were also obtained when examining only doubly and only triply charged peptides separately. These results can be found in the Supporting Information (Sheets S1–S4). Furthermore, we compared the heights of the two peaks in the score vs collision energy curves of bimodal peptides, and we found that, in most of the cases, i.e., for 80–90% of the peptides depending on the sample and search engine, the lower energy peak provides higher scores (see details in Tables S4 and S5). Therefore, the unimodal optimum and the lower energy optimum of the bimodal peptides were used together to determine the CE settings for digestions with Asp-N, chymotrypsin, and Glu-C.

### Comparison of Different Samples and Search Engines

We investigated the effect of sample type and applied search engine, and it was shown that the optimal CEs of peptides do not depend on these factors (see Figures S2–S4). Based on these findings, only plasma-related results evaluated with Mascot will be presented in detail hereafter (for more results, see Sheet S5).

### Comparison of Different Enzymes

**Figure 3:**
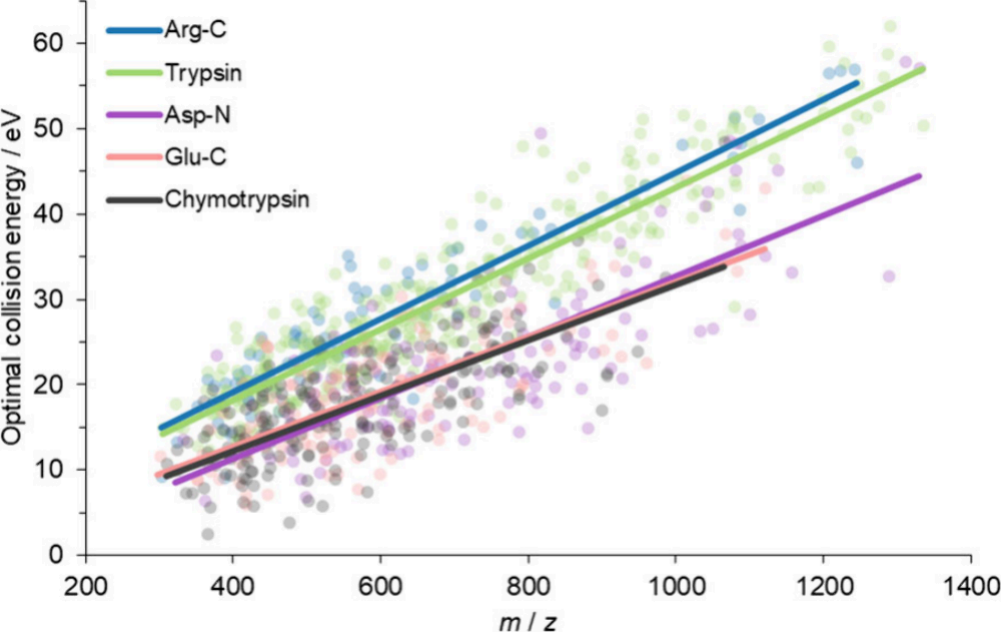
Influence of the enzyme type. Optimal CE settings in eV as a function of *m*/*z* for doubly charged peptides digested by Arg-C (blue), trypsin (pale green), Asp-N (purple), Glu-C (pastel pink), and chymotrypsin (gray) from human blood plasma, using Mascot search engine. Solid lines represent linear fits of the measured data points and highlight the distinct linear trend for trypsin and Arg-C compared to the other enzymes.

For the enzyme dependence of optimal CE settings, we compared the optimal CE settings of different alternative enzymatic digestions. The results of trypsin and Arg-C are practically the same, and the fitted lines do not differ significantly from each other as exemplified in Figure 3 for human blood plasma digest with the Mascot search engine. This can be explained by the structural similarity of Arg-C and tryptic peptides, i.e., having basic amino acid at the C-terminal. Then, we compared the digests of Asp-N, Glu-C, and chymotrypsin to trypsin. The CE dependences of peptides resulting from the use of the three alternative enzymes are very similar to each other, and the data nearly overlap (see Figure 3). On the other hand, these are very different from those of tryptic and Arg-C peptides. More precisely, the optimized CE settings show a significant difference: the peptides produced by Asp-N, Glu-C, and chymotrypsin enzymes fragment at a collision energy 5–15 eV lower than tryptic (and Arg-C) digestions. In other words, peptides produced by Glu-C, Asp-N, and chymotrypsin require ca. 20% lower CE setting for fragmentation.

**Figure 4:**
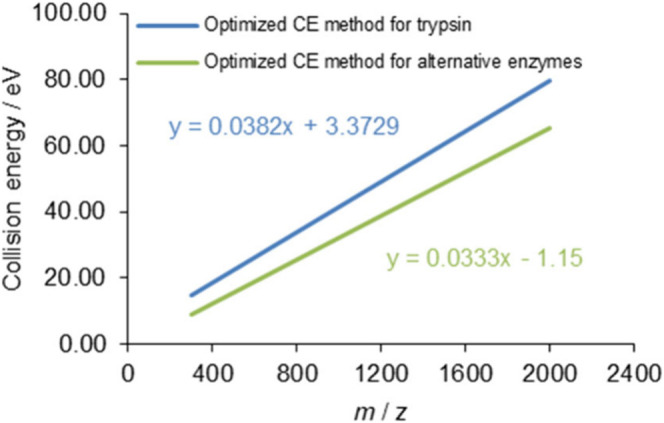
Comparison of optimized collision energy settings in eV as a function of *m*/*z* for +2 peptides (results for +3 peptides can be found in the SI). Blue line represents the tryptic (and Arg-C) optimized CE setting,^34^ while the green line represents the optimized CE setting for Glu-C, Asp-N, and chymotrypsin. For both charges, these three enzymes require a lower collision energy setting.

Based on these results, samples digested by Arg-C can be measured using the same MS/MS CE settings optimized for tryptic peptides, while it is advantageous to use lower values (by about 20%) for samples digested by Glu-C, Asp-N, and chymotrypsin. Therefore, we determined optimized parameters using the combined Mascot results obtained from the investigation of these three enzymatic digests from human blood plasma results (neither the search engine nor the sample affects the results see Comparison of Different Samples and Search Engines). According to our experience that the optimal collision energy depends on the charge, we determined separate CE settings for doubly and triply charged peptides in the same way as for tryptic peptides^34^ (see Figure 4 and Figure S5).

On an Orbitrap instrument, the determined optimal experimental MS/MS settings belong to 22–27 NCE% in the case of trypsin and Arg-C depending on the *m*/*z* value and charge state, while optimal CEs fall in the range of 16–22 NCE% in the case of Glu-C, Asp-N, and chymotrypsin (for more information see Material S4 and Table S6).^51^ The NCE% calculated for trypsin and Arg-C coincides with the value calculated for the previous tryptic peptides from Hela and *E. coli*, where 20–29% were obtained.^38^ We determined that the alternative enzymatic peptides require ca. 20% lower CE setting for fragmentation. On an Orbitrap instrument, it would belong to smaller NCE% by ca. 5–6 NCE%.

A recent study investigated 4 NCE% values (24%, 27%, 30%, and 33%) for trypsin, Glu-C, and Asp-N to optimize the data-independent acquisition method using HEK 293T lysate.^10^ In their case, the same settings (27 and 30 NCE%) were found to be optimal for all enzymes. Although our results agreed regarding the number of b- and y- ions, see Relationship between Optimal CEs and Fragmentation Characteristics, their optimal collision energy results differ from ours; however, we conducted a more comprehensive examination of the collision energy, measuring a total of 21 collision energy values, not just 4. We have no information about whether any repeated measurements were performed in their case. Furthermore, we used a device different from that used by them, which could also have contributed to this contradiction. Another key difference is that they optimized a multiprotease DIA method and DDA and DIA have distinct fragmentation strategies. The effects of CE settings observed in DDA measurements can be mitigated by the DIA windows, where peptides with multiple charge states are often fragmented simultaneously. DIA analyses also require spectral libraries and deconvolution software, which can account for relative fragment intensities with greater precision compared to search engines like Mascot and Byonic. Software for in silico library generation in DIA experiments can integrate CE effects during spectra generation, potentially compensating for suboptimal fragmentation conditions.^52^

### Investigation of the Performance of the Determined CE Setting

Performance tests were conducted with human blood plasma digested by Arg-C, chymotrypsin, Glu-C, and trypsin and examined at two collision energy settings: (1) at the collision energy method previously optimized for tryptic peptides of HeLa and *E. coli*(34) and (2) our optimized collision energy setting for nontryptic peptides. The latter optimized setting was collectively established for peptides produced by Asp-N, chymotrypsin, and Glu-C. During the experiments, all digests were measured with 3 repetitions at both MS settings, and data were evaluated using both Byonic and Mascot.

To determine the performance gain between the two CE methods, we compared the results of human blood plasma digested with the same enzyme but measured with different collision energy settings. This allowed us to assess whether more peptide hits and higher score values were obtained with the CE method optimized for alternative enzymes or for trypsin. We drew the same conclusions for both database search engines. No difference was found in protein hits, but there was a difference in the number of peptide hits. For chymotrypsin and Glu-C, the average number of peptide hits of the parallel measurements increased by 23–32% with Mascot and by 8–14% with Byonic in favor of the CE method optimized for alternative enzymes. However, for Arg-C and trypsin, the tryptic optimal CE method was better with an increase of 14–14% with Mascot and 6–7% with Byonic. Exact numbers of peptide hits can be found in Supporting Information Table S7.

Since no significant difference was observed at the protein level but differences were found in the number of peptides, we examined the change in sequence coverage in the case of the two methods (3 repetitions in both cases). We investigated the proteins that were identified at least once using both CE methods. For these proteins, we divided the length of the entire protein sequence by the length of the identified protein sequence. The calculated sequence coverages for these proteins were averaged per measurement, and finally, the repetitions for the given collision energy setting were averaged. In the case of trypsin and Arg-C, the sequence coverage was 7% and 12% higher with the tryptic CE method, respectively. For Glu-C and chymotrypsin, sequence coverage increased by 15% and 16% on average, when using the optimal CE for alternative enzymes (see Table S8 for further details). The number of peptide hits and the sequence coverage proved that, while the optimized CE method for trypsin is better for Arg-C and trypsin, our new optimized CE method for alternative enzymes is better for chymotrypsin and Glu-C.

**Figure 5:**
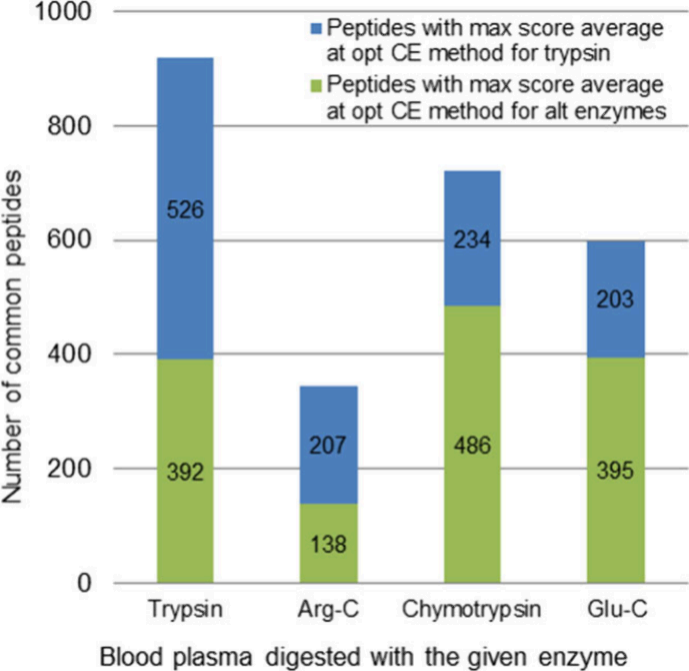
Number of peptides that were identified at least once by both optimized CE methods from human blood plasma for a given enzyme with Mascot. Blue bars show how many peptides have a higher average score value with the optimized CE method for trypsin, while green bars show how many peptides have a higher average score value with the CE method optimized for alternative enzymes.

Regarding the identification scores, the trends were the same for the two database search engines. There was no significant difference between the averages of the score values of all of the peptides identified in the given measurements. However, at the peptide level, we found an improvement in the score values. The score differences were examined for the peptides that were identified at least once by both CE methods for a given enzyme. If the peptides were identified multiple times in parallel measurements for a given CE method, then we averaged the score values. Then, we compared the score values obtained by the two CE methods for a given peptide and determined which method yielded a higher score for the specific peptide. This way, we were able to determine at the peptide level which optimized CE method provided a better identification score for a given enzyme. For Arg-C and trypsin, ca. 60% of the peptides identified had higher average score values with the optimized method for trypsin with Mascot, while for chymotrypsin and Glu-C, 75% and 67% of the peptides received higher average scores with the CE method optimized for alternative enzymes, respectively (see Figure 5.). Similar values were obtained when we only looked for the maximum score values, and we also obtained the same trends with Byonic in both cases (Figures S6 and S7).

### Relationship between Optimal CEs and Fragmentation Characteristics

**Figure 6:**
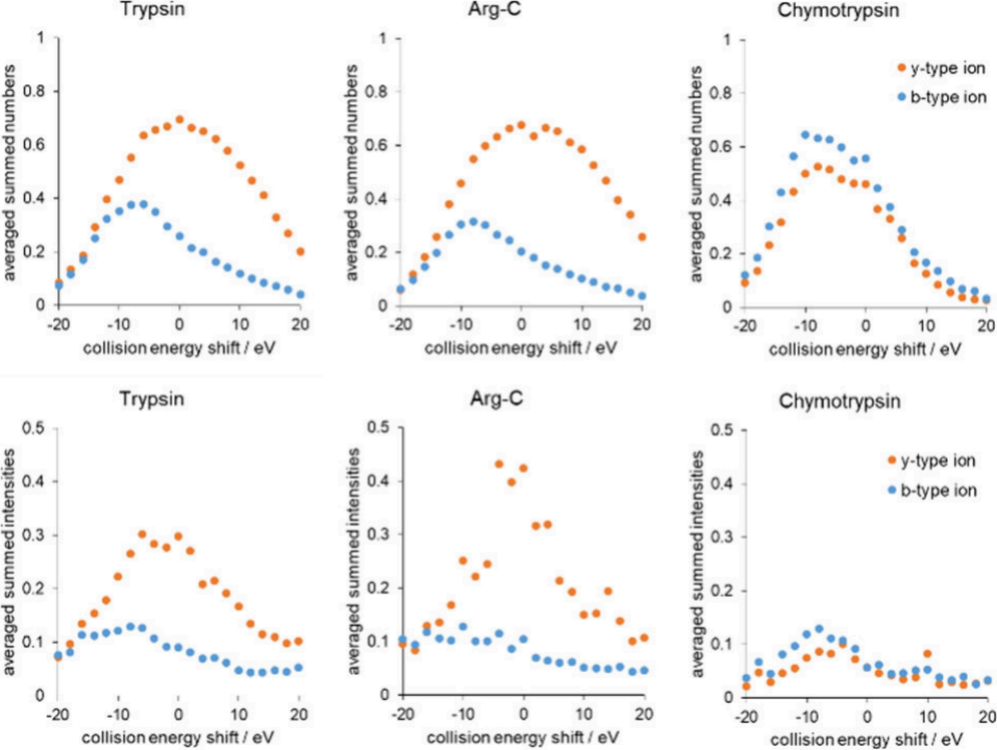
Average summed numbers (top) and intensities (bottom) of b- and y-ions of human blood plasma digested by trypsin (left), Arg-C (middle), and chymotrypsin (right) as a function of collision energy shift. y-ions are marked by orange circles, while b-ions are blue circles.

In order to understand and find an explanation for the differences between the optimal CE trends of peptides obtained by enzymatic digestion using various enzymes, the summed number and intensity of b- and y-ions were investigated as a function of the collision energy obtained from the Mascot .dat output files (see Figure 6). For this purpose, all peptides analyzed during the energy-dependent measurement series were involved in this study. To map trends and not focus on individual peptides, we averaged the values for all peptides at the given CE shifts. Since the optimal collision energy depends linearly on *m*/*z*, we did not use the absolute value of the collision energy but the extent of the deviation from the fitted line representing the tryptic optimum value (shift value of 0).

The intensities of y- and b-ions were all divided by their precursor ion intensities before averaging in order to consider the individual peptides with the same weight. In the case of the number of b- and y-ions, they were divided by their maximum possible value coming from the peptide length.

In the case of trypsin, our results are in line with the literature, namely, y-type ions are more numerous and more intense at all CE settings than b-type fragments; their maximum is at 0 CE shift, and their relative abundance also increases with the collision energy (see Figure 6 left).^10,32,34,35^ Regarding the average summed intensities and numbers of the fragments of peptides prepared by Arg-C (Figure 6 middle), y-ions are present in greater numbers than b-ions, in the same way as with trypsin. It can also be observed that the number of y-ions of tryptic and Arg-C peptides have maximums at the collision energy shift value of 0, which is not surprising, because this is the collision energy optimized for tryptic peptides.

On the other hand, in the case of Asp-N, Glu-C, and chymotrypsin, both the intensities and numbers of y- and b-ions are almost the same regardless of collision energy, and even the b ions are more intense/numerous (Figure 6 right and Sheet S6). The previously mentioned study on an Orbitrap instrument found similar fragment ion trends tested for 24, 27, 30, and 33 NCE% values for Glu-C and Asp-N using data-independent acquisition.^10^ Furthermore, in the case of these alternative proteolytic enzymes, the maximum intensity and number of both b- and y-ions reached well below the tryptic optimum by ca. 8–10 eV. All this coincides with the fact that a collision energy setting 5–15 eV lower gives the most reliable identification for these enzymes.

Peptides produced during tryptic digestion have lysine or arginine at the C-terminal. In this case, y-ions are relatively stable because the basic amino acids at the C-terminus stabilize them. This stabilization occurs because the positive charge is preferentially localized on the basic residue of C-terminal.^35^ b-ions are less stable than y-ions, and accordingly, MS/MS spectra of tryptic peptides are characterized by intense y-series, while b-type ions are typically only found in smaller numbers and intensities in the spectra,^31−34^ as also observed in our case with trypsin and Arg-C (see Figure 6 and Sheet S6). This phenomenon is not experienced with Asp-N, Glu-C, and chymotrypsin since they cleave only at a nonbasic amino acid. Thus, in these cases, the basic amino acids are located randomly in the peptide chain, and so, the intensities of y- and b-ions are comparable. All of these suggest that there is no significant difference in the stability of b- and y-ions when these three alternative proteolytic enzymes are used for digestions.

**Figure 7:**
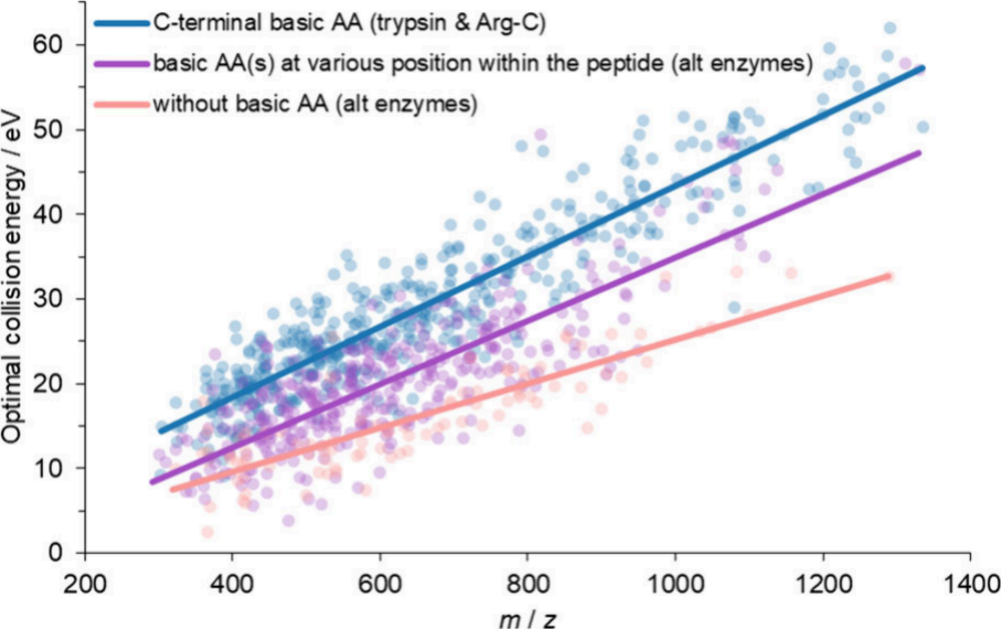
Influence of basic amino acids on the optimal collision energy of peptides. Optimal collision energies in eV as a function of *m*/*z*. Peptides from trypsin and Arg-C, which have basic amino acids (AA) at the C-terminal, are marked by blue points. Peptides from Asp-N, Glu-C, and chymotrypsin (alt enzymes) containing basic amino acid(s) at various position(s) within their sequences are marked by purple points. Peptides from Asp-N, Glu-C, and chymotrypsin without basic amino acid in their sequences are marked by pastel pink.

Regarding the influence of basic amino acids on the optimal collision energy, we compared peptides from trypsin and Arg-C, which have basic amino acid at the C-terminal; peptides from Asp-N, Glu-C, and chymotrypsin, which contain basic amino acid(s) at various position(s) within their sequences; and peptides from Asp-N, Glu-C, and chymotrypsin without basic amino acids in their sequences (see Figure 7). We observed that basic amino acid at the C-terminal stabilizes the peptide, resulting in higher optimal collision energy. Peptides with basic amino acid(s) at random positions within the peptide sequence show lower optimal collision energy, while peptides that do not contain any basic amino acid have the lowest optimal collision energy. Based on these results, the presence and the position of basic amino acids plays a significant role in the optimal collision energy of peptides. Therefore, optimal collision energy methods can be chosen based on whether the proteolytic enzyme generates peptides with basic amino acid at the C-terminal.

## Conclusions

During the past decade, alternative enzymes having various cleavage specificities have gained more and more attention in the field of bottom-up proteomics. Their use–either alone or in combination with trypsin–can provide more insight into the proteome of biological samples and can be advantageous in several specific applications. On the other hand, mass spectrometry-based workflows of peptide identifications have been typically set out with trypsin and therefore may be suboptimal for alternative enzymatic digests. In the present project, we focused on the collision energy value applied during the CID process of DDA experiments, as this parameter has major influence on the information content of the MS/MS measurement. We examined the CE-dependent fragmentation of several thousand peptides produced by various widely used enzymes, determined the peptide level optimal CE setting from confident identification point of view, and proposed an enzyme-dependent CE method. The main conclusions that we could draw from our work are as follows.

- The fragmentation behavior and optimal CE for tryptic and Arg-C peptides are practically the same providing nearly identical trendlines for the CE method. This can be caused by the similar physicochemical properties caused by the basic amino acid at the C-terminal, i.e., R for Arg-C and R or K for trypsin.
- The CE dependence of peptides resulting from the use of Glu-C, Asp-N, and chymotrypsin are very similar to each other; on the other hand, these are very different from those of tryptic and Arg-C peptides. The trendlines belonging to the optimized CE settings fall at a collision energy 5–15 eV lower than for tryptic (and Arg-C) digestions on our Bruker QTof instrument. It means that these peptides require ca. 20% lower CE setting for fragmentation. On an Orbitrap instrument, it would belong to smaller NCE% by ca. 5–6 NCE%.
- The results are analogous for both samples investigated and for both database search engines applied.
- The proposed CE method based on our peptide level results resulted in significantly more peptide hits by 23–32% with Mascot and by 8–14% with Byonic depending on the alternative enzyme. The sequence coverage of the identified proteins and the confidence of peptide spectrum matches (characterized by the score values) also increased. Therefore, a CE setting lower by ca. 20% for alternative enzymes is clearly advantageous.
- The different behavior of peptides is in line with their different fragmentation characteristics: the y-ions for Arg-C and tryptic peptides are significantly more stable than in the case of other enzymes due to the stabilization effect of R/K amino acids present. In the case of alternative enzymes, peptides with basic amino acid(s) at random positions within the peptide sequence show lower optimal collision energy, while peptides that do not contain any basic amino acid have the lowest optimal collision energy. So, the position of basic amino acid plays a significant role in the optimal collision energy of peptides. Optimal collision energy methods can be chosen based on whether the proteolytic enzyme generates peptides with basic amino acid at the C-terminal.

We believe that our data show insight into the different fragmentation properties of peptides from different enzymatic digests and provide a solid background to a more efficient MS/MS CE choice for researchers using any mass spectrometric platform. Additionally, in tandem mass spectrometry, machine learning tools are increasingly used for spectrum identification and prediction across diverse molecular types.^53,54^ Accordingly, such applications have also emerged in MS-based proteomics.^52,55−57^ Our data, measured at different collision energies with varying enzyme specificities and our findings regarding the b- and y-ions of alternative enzymes may support the development of machine learning models or further refine existing ones for nontryptic peptides.

## Supporting Information Available

Table S1: Different Parameters of Enzymatic Digestions; Material S1: Details of Nano-LC-MS/MS Measurements; Material S2: Peptide Identification Using Byonic and Mascot Search Engines; Material S3: Determination of Optimal Collision Energy Setting Using Serac; Figure S1: Score vs CE Curves for ATWSGAVLAGR^2+^ with Byonic and Mascot; Table S2: Number of Identified Peptides from Human Blood Plasma Digested by Different Proteolytic Enzymes; Table S3: Number of Identified Peptides from Human Protein Extract Digested by Different Proteolytic Enzymes; Table S4: Percentages of Bimodal Peptides when Lower CE Provided Higher Score Than Higher CE in the Case of Human Plasma; Table S5: Percentages of Bimodal Peptides when Lower CE Provided Higher Score Than Higher CE in the Case of Human Protein Extract; Figure S2: Influence of Sample Type on Optimal CE; Figure S3: Peak Positions of Hela and *E. coli* with Plasma and Human Protein Extract; Figure S4: Comparison of Different Samples and Search Engines; Figure S5: Optimized CE Methods for +3 Charged Peptides; Material S4: Calculation of Optimized CE Setting from eV to NCE%; Table S6: Calculated Optimized CE Settings in NCE%; Table S7: Exact Numbers of Peptide Hits during Performance Tests; Table S8: Sequence Coverages during Performance Tests; Figure S6: Higher Average Scores for Peptides with Byonic; Figure S7: Maximum Scores for Peptides with Byonic and Mascot (PDF)
Collision energy trends for Mascot and Byonic data (XLSX)
