# Supplementary material for: The Enzyme Effect: Broadening the Horizon of MS Optimization to Nontryptic Digestion in Proteomics
Source: J Am Soc Mass Spectrom. 2025 Jan 13;36(2):299–308. doi: 10.1021/jasms.4c00396 (PMC11808764; doi:10.1021/jasms.4c00396)
Supplement: Supplementary file 1 — js4c00396_si_001.pdf [file js4c00396_si_001.pdf]

Supporting information:

The enzyme effect: Broadening the horizon of MS optimization to non-tryptic digestion in proteomics

Kinga Nagy,[1,2] Péter Sándor,[1] Károly Vékey,[1] László Drahos,[1] Ágnes Révész*[1]

[1] MS Proteomics Research Group, HUN-REN Research Centre for Natural Sciences, Magyar Tudósok körútja 2., H-1117, Budapest, Hungary

[2] Hevesy György PhD School of Chemistry, ELTE Eötvös Loránd University, Faculty of Science, Institute of Chemistry, Pázmány Péter sétány 1/A, Budapest, H-1117, Hungary

e-mail: revesz.agnes@ttk.hu

Table of Contents

Further Experimental Details

Table S1: Different Parameters of Enzymatic Digestions.

Material S1: Details of Nano-LC-MS/MS Measurements.

Material S2: Peptide Identification Using Byonic and Mascot Search Engines.

Material S3: Determination of Optimal Collision Energy Setting Using Serac.

Figure S1: Score vs. CE Curves for ATWSGAVLAGR^{2+} with Byonic and Mascot.

Table S2: Number of Identified Peptides from Human Blood Plasma Digested by Different Proteolytic Enzymes.

Table S3: Number of Identified Peptides from Human Protein Extract Digested by Different Proteolytic Enzymes.

Table S4: Percentages of Bimodal Peptides when Lower CE Provided Higher Score Than Higher CE in the Case of Human Plasma.

Table S5: Percentages of Bimodal Peptides when Lower CE Provided Higher Score Than Higher CE in the Case of Human Protein Extract.

Figure S2: Influence of Sample Type on Optimal CE.

Figure S3: Peak Positions of Hela and *E. Coli* with Plasma and Human Protein Extract.

Figure S4: Comparison of Different Samples and Search Engines.

Figure S5: Optimized CE Methods for +3 Charged Peptides.

Material S4: Calculation of Optimized CE Setting from eV to NCE%.

Table S6: Calculated Optimized CE Settings in NCE%.

Table S7: Exact Numbers of Peptide Hits during Performance Tests.

Table S8: Sequence Coverages during Performance Tests.

Figure S6: Higher Average Scores for Peptides with Byonic.

Figure S7: Maximum Scores for Peptides with Byonic and Mascot.

Further Experimental Details

Table S1: Different Parameters of Enzymatic Digestions (trypsin, Arg-C, Asp-N, chymotrypsin and Glu-C).

Enzyme	Digestion time	Digestion temperature (°C)	Protein:enzyme ratio (wt/wt)*[2]
**Trypsin**	Lys-C/trypsin mixture: 1 h<br>Trypsin: 2 h	37	LysC-Trypsin mixture: 100:1<br>Trypsin: 25:1
**Arg-C***	3 h	37	20:1
**Asp-N**	12 h	37	75:1
**Chymotrypsin, Glu-C**	12 h	25	75:1

*In the case of Arg-C, since alkylating reagents are inhibitors of Arg-C, before adding the enzyme, $CaCl_2$ and DTT were added to the sample (10 min).

*[2] For the determination of protein:enzyme ratio, the concentration of blood plasma was determined with Nanodrop ND-1000 spectrophotometer.

Material S1: Details of Nano-LC-MS/MS Measurements

Liquid chromatography-mass spectrometry investigations were carried out on a Bruker Maxis II ETD Q-TOF (Bruker Daltonics, Bremen, Germany) mass spectrometer equipped with a CaptiveSpray nanoBooster ionization source coupled to an Ultimate 3000 RSLCnano System (Dionex, Sunnyvale, CA, USA) under the control of Hystar v. 3.2 (Bruker Daltonics, Bremen, Germany). In each run, 2-2.25 µg enzymatic digest of human protein extract or 1 µg enzymatic digest of blood plasma was injected onto an Acclaim PepMap 100 C-18 trap column (5 µm, 100 Å, 100 µm × 20 mm, Thermo Fisher Scientific, Waltham, MA, USA) using 0.1% trifluoroacetic acid (TFA). Peptides were separated on an Acquity M-Class BEH130 C18 analytical column (1.7 µm, 130 Å, 75 µm ×250 mm Waters, Milford, MA) in case of the energy dependent studies and performance check experiments. Temperature was set at 48 °C and a flow rate of 300 nl/min was applied for energy dependent studies and performance check, respectively. Solvents A and B were 0.1% FA in water and in ACN, respectively. The applied gradient program was as follows: solvent B content was increased from 4 to 25% in 75 min, then to 40% in 15 min and 90% in 1 min, washed for 5 min, and finally the column was equilibrated with 4% B for 20 min.

Sample ionization was achieved in the positive electrospray ionization mode via a CaptiveSpray nanoBooster ion source. The capillary voltage was set to 1360 V, the nanoBooster pressure was 0.3 bar, the drying gas was heated to 180 °C, and the flow rate was 3 l/min. Internal mass calibration was performed via lock mass for each run using sodium formate according to Brukers' recommendation: 1 mmol sodium formate in 15% MeOH solution were infused at a flow rate of 0.03 ml/h into Acclaim PepMap 100 C-18 trap column (5 µm, 100 Å, 100 µm × 20 mm, Thermo Fisher Scientific, Waltham, MA, USA). The ion transfer parameters were set as follows: prepulse storage 10 µs, quadrupole ion energy 5 eV, Funnel 1 RF 400 Vpp, Multipole RF 400 Vpp. The collision RF was set to 1200 Vpp, and the ion transfer time was 120 µs. For the MS measurements, a fix cycle time of 2.5 sec was used. MS spectra were acquired over a mass range of 150–2200 m/z at 3 Hz, while CID was performed at 16 Hz for abundant precursors and at 4 Hz for ones of low abundance.
The collision energy was determined by the control software based on the m/z value and charge of the precursor ion. For mass calibration, data was recalibrated by the Compass DataAnalysis software 4.3 (Bruker Daltonics, Bremen, Germany).

Material S2: Peptide Identification Using Byonic and Mascot Search Engines

Byonic search

We employed Byonic v5.0.20 for our analyses (Protein Metrics, Cupertino, CA). The measurements were evaluated using human SwissProt (August 2022). The specific cleavage sites of the enzymes were: N-terminal of aspartic acid (D) and glutamic acid (E) for Asp-N, C-terminal of arginine (R) for Arg-C, C-terminal of aspartic acid (D) and glutamic acid (E) for Glu-C, C-terminal of phenylalanine (F), tryptophan (W), tyrosine (Y), leucine (L), methionine (M), alanine (A), aspartic acid (D) and glutamic acid (E) for chymotrypsin, C-terminal of arginine (R) and lysine (K) for trypsin. Cleavage specificity was set as semi specific for chymotrypsin and Glu-C based on recommendations of Preview module and personal experiences, and fully specific for the other enzymes. Regarding mass tolerance values, recommendations of the Preview module were used: 10 and 20 ppm for precursor and fragment mass tolerance for human protein extract samples, and 20-20 ppm for precursor and fragment mass tolerance for human blood plasma samples. As fixed modification, carbamidomethylation (C) was selected. The variable modifications were oxidation (M) as common and pyro-glutamination (N-term Q and N-term E), ammonia loss (N-term C), acetyl loss (protein N-term) and deamidation (N and Q) as rare modifications.

Mascot search

MS/MS peak list generation was performed using ProteinScape software 4.0.3 (Bruker Daltonik GmbH, Bremen, Germany). Database search was performed using the Mascot search engine version v.2.5.1 (Matrix Science, London, U.K.). The measurements were evaluated using human SwissProt (August 2022). The mass tolerance was set as 10 ppm for precursors of human protein extract and 20 ppm for precursors of human blood plasma digests. The mass tolerance for fragment peaks was set as 0.05 Da for human protein extract and 0.1 Da for human blood plasma digests, respectively. Fixed modification was carbamidomethylation (C), and the variable modifications were oxidation (M) and deamidation (N and Q).

Material S3: Determination of Optimal Collision Energy Setting Using Serac[1]

The pre-optimized *default collision energy setting* is given by the following equation[1]:

collision energy (eV) = 0.0368 × (precursor m/z) + 4.2786.

Further measurements at values higher and lower than this setting, from −20 eV to +20 eV, in 2 eV steps were calculated from this equation.

For the determination of optimal collision energy, the score versus collision energy shift functions were first normalized by dividing all points with the maximum score for the given peptide ion. For each peptide ion, the optimum energy was determined from the normalized score versus collision energy shift data sets by fitting one or two Gaussian functions. As we did not have any data points with score below 100 and 15 for Byonic and Mascot search engine, respectively, we decided to add two points with zero score at a shift of ± 35 eV. Two Gaussian fits were accepted if all of the following conditions were met:

a) sum of squares of residuals for the two-peak fit was less than 80% of that of the one-peak fit,
b) neither of the two peaks was exceedingly narrow (e.g., fitted to a single outlier data point),
c) neither of the two peaks had its maximum outside the actually measured collision energy shift range
d) the maximum value of the fitted curve was less than 1.2.

If these conditions were not met, optimal collision energy was obtained from the one Gaussian fit.

(1) Révész, Á.; Rokob, T. A.; Jeanne Dit Fouque, D.; Turiák, L.; Memboeuf, A.; Vékey, K.; Drahos, L. Selection of Collision Energies in Proteomics Mass Spectrometry Experiments for Best Peptide Identification: Study of Mascot Score Energy Dependence Reveals Double Optimum. J. Proteome Res.2018, 17, 1898–1906. https://doi.org/10.1021/acs.jproteome.7b00912

Score vs. CE Curves for ATWSGAVLAGR^{2+} with Byonic and Mascot

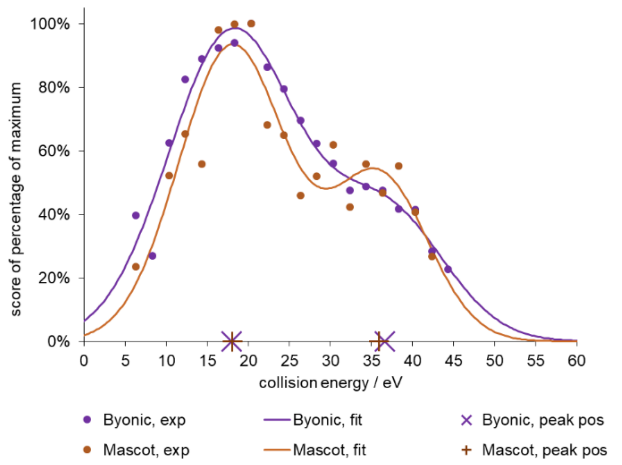

Figure S1: Experimental points together with the fitted Gaussian functions for all investigated identification scores for the example peptide ATWSGAVLAGR^{2+} from tryptic digestion of human blood plasma. Symbols denote measured data, while solid lines depict the model functions. The peak positions of the latter are marked by crosses on the horizontal axis. Purple: Byonic; Brown: Mascot.

Table S2: Number of Identified Peptides from Human Blood Plasma Digested by Different Proteolytic Enzymes. Regarding the human blood plasma samples, Byonic search engine provides more peptide hits in the case of trypsin and Arg-C, while Mascot search engine is more efficient for Glu-C, chymotrypsin and Asp-N. The same trend holds true for the number of peptides considered in the CE analysis, except for Glu-C, where interestingly we could involve more species using Byonic. The results for human protein extract show similar trends with respect to the comparison of search engine performance (see Table S3). The charge state distribution of the peptides selected for CE dependence were similar for the two sample types and search engines. For trypsin, Arg-C and Asp-N approximately half of the peptides were doubly charged and one third were triply charged. In the case of chymotrypsin and Glu-C, the portion of species having +2 charge is slightly larger (66–86%), whereas 20–10% is in charge state +3. Note that the number of peptides with +4, +5 or +6 charge were too small for drawing reliable conclusions, therefore we will not discuss them separately.

Human Blood Plasma		Mascot		Byonic	
		Unimodal	Bimodal	Unimodal	Bimodal
Trypsin	Identified from all runs (>25 or >300 score)	1588		1680	
	Considered for energy dependence study	984		1439	
	From this, 2+	362	198	312	460
	From this, 3+	224	105	262	250
Arg-C	Identified from all runs (>25 or >300 score)	450		452	
	Considered for energy dependence study	279		393	
	From this, 2+	94	51	60	122
	From this, 3+	67	29	80	67
Glu-C	Identified from all runs (>25 or >300 score)	515		386	
	Considered for energy dependence study	189		280	
	From this, 2+	103	22	131	55
	From this, 3+	29	8	40	24
Chymotrypsin	Identified from all runs (>25 or >300 score)	738		375	
	Considered for energy dependence study	228		201	
	From this, 2+	170	27	118	55
	From this, 3+	19	4	20	6
Asp-N	Identified from all runs (>25 or >300 score)	494		312	
	Considered for energy dependence study	315		281	
	From this, 2+	98	37	63	84
	From this, 3+	91	19	48	39

Table S3: Number of Identified Peptides from Human Protein Extract Digested by Different Proteolytic Enzymes.

Human protein		Mascot		Byonic	
		Unimodal	Bimodal	Unimodal	Bimodal
Trypsin	Identified from all runs (>25 or >300 score)	5592		4408	
	Considered for energy dependence study	2803		3456	
	From this, 2+	1091	324	957	797
	From this, 3+	749	247	834	443
Glu-C	Identified from all runs (>25 or >300 score)	1071		729	
	Considered for energy dependence study	323		502	
	From this, 2+	221	23	289	92
	From this, 3+	57	9	68	26
Chymotrypsin	Identified from all runs (>25 or >300 score)	1185		339	
	Considered for energy dependence study	354		185	
	From this, 2+	234	26	143	29
	From this, 3+	61	8	11	2
Asp-N	Identified from all runs (>25 or >300 score)	698		185	
	Considered for energy dependence study	238		132	
	From this, 2+	104	6	82	12
	From this, 3+	49	8	21	4

Table S4: Percentages of Bimodal Peptides when Lower CE Provided Higher Score Than Higher CE in the Case of Human Plasma.

	2+ – 6+		2+		3+	
	Byonic	Mascot	Byonic	Mascot	Byonic	Mascot
Trypsin	93.89%	80.00%	97.39%	76.77%	88.40%	85.71%
Arg-C	91.00%	86.05%	97.54%	86.27%	86.57%	86.21%
Asp-N	91.24%	91.04%	90.48%	94.59%	97.44%	89.47%
Chymotrypsin	93.44%	77.42%	94.55%	81.48%	83.33%	50.00%
Glu-C	92.05%	81.58%	92.73%	95.45%	87.50%	62.50%

Table S5: Percentages of Bimodal Peptides when Lower CE Provided Higher Score Than Higher CE in the Case of Human Protein Extract.

	2+ – 6+		2+		3+	
	Byonic	Mascot	Byonic	Mascot	Byonic	Mascot
Trypsin	94.39%	79.82%	98.37%	77.16%	89.62%	86.23%
Asp-N	77.78%	72.00%	83.33%	83.33%	75.00%	62.50%
Chymotrypsin	96.77%	88.57%	96.55%	88.46%	100.00%	100.00%
Glu-C	93.70%	97.22%	94.57%	100.00%	96.15%	88.89%

Influence of Sample Type on Optimal CE

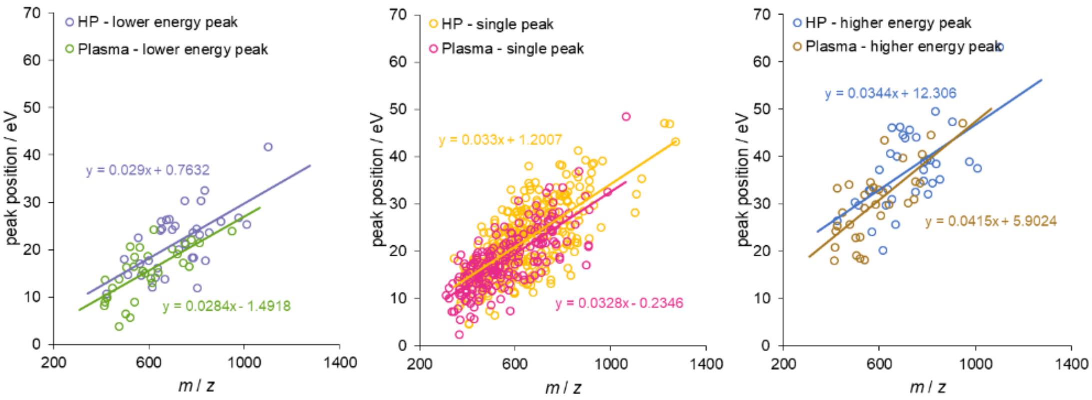

Figure S2: Peak positions in eV as a function of *m/z* for peptides with charge states from +2 to +6 produced by chymotrypsin, using Mascot search engine. Yellow (human protein extract) and reddish pink (human blood plasma) circles indicate the position of the sole peak for peptides having unimodal behavior, while blue (human protein extract), light brown (human blood plasma), violet (human protein extract) and green (human blood plasma) circles are the higher and the lower collision energies, respectively, for bimodal peptides. Solid lines represent linear fits of the measured data points. The fitted lines are very similar and give approximately the same result with peptides produced by chymotrypsin from human blood plasma and human protein extract. We also experienced this with the other enzymes using both search engines, to a greater or lesser extent.

Peak Positions of Hela and *E. coli* with Plasma and Human Protein Extract

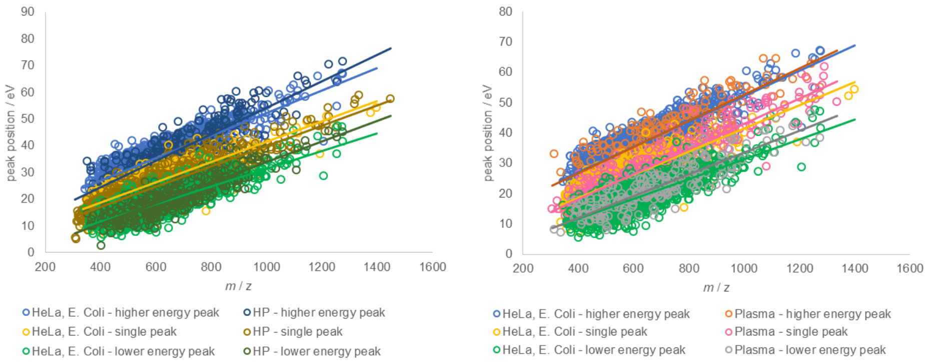

Figure S3: Peak positions in eV as a function of *m/z* for peptides with +2 charge state produced by trypsin, using Mascot search engine. The obtained trends for previous results of tryptic HeLa and *E. coli* samples were compared to obtained trends of tryptic peptides of human protein extract (see left) and human blood plasma (see right). The fitted lines are very similar and give approximately the same result, which shows that the type of sample has negligible effect on the optimal CEs of peptides.

Comparison of Different Samples and Search Engines

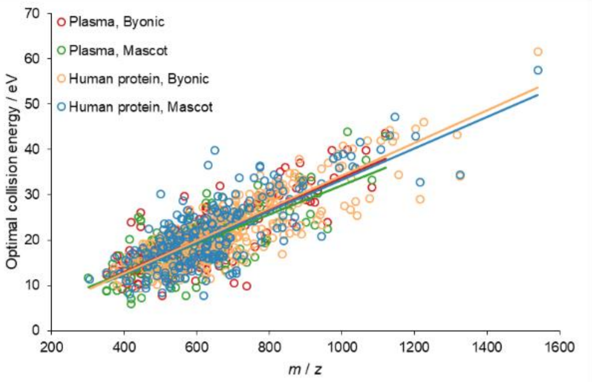

Figure S4. Influence of search engine and sample type on the optimal CE setting. Compared optimal CE settings in eV as a function of *m/z* for doubly charged peptides produced by Glu-C, using Mascot and Byonic search engines. Red (Byonic) and green (Mascot) circles belong to peptides from human blood plasma, respectively. Light orange (Byonic) and light blue (Mascot) circles depict peptides from human protein extract, respectively. Solid lines represent linear fits of the measured data points. The optimal CE settings for doubly charged peptides from human blood plasma and human protein extract digested with Glu-C fall on the same line. It also shows that the optimal CE setting does not depend much on the database search engine applied for data analysis (Byonic and Mascot). The optimal CE settings are not affected by the search engine and sample type used.

Optimized CE Methods for +3 Charged Peptides

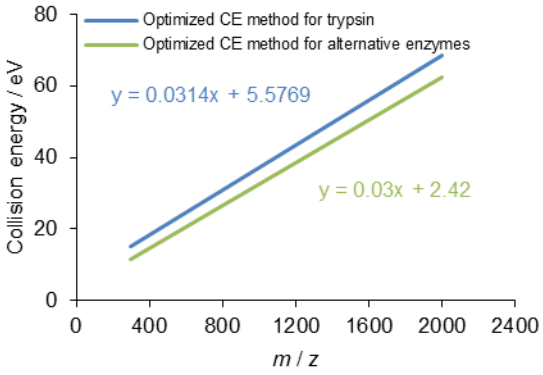

Figure S5: Compared optimized collision energy settings in eV as a function of *m/z* for +3 peptides. Blue line represents the tryptic (and Arg-C) optimized CE setting, while green line represents the optimized CE setting for Glu-C, Asp-N and chymotrypsin. For both +2 and +3 charges, these three enzymes require a lower collision energy setting.

Material S4: Calculation of Optimized CE Setting from eV to NCE%[2,3]

The calculation of eV to NCE% depends on the m/z value and charge state:

collision energy (eV) = NCE (%) × (precursor m/z) / 500 × (charge factor)

where charge factor is 0.9 for +2 and 0.85 for +3 charge states.

The determined optimized settings in NCE% were calculated by this equation. The exact values can be found in Table S6.

(2) News in Proteomics Research: Normalized collision ener-gy calculation for Q Exactive https://proteomicsnews.blogspot.com/2014/06/normalized-collision-energy-calculation.html (accessed Mar 28, 2024)

(3) Révész Á., Milley M. Gy., Nagy K., Szabó D., Kalló G., Csősz É., Vékey K., Drahos L. Tailoring to Search Engines: Bottom-Up Proteomics with Collision Energies Optimized for Identification Confidence. Journal of Proteome Research 2021 20 (1), 474-484 https://doi.org/10.1021/acs.jproteome.0c00518

Table S6: Calculated Optimized CE Settings in NCE%. The calculated optimal CE settings in eV and NCE% of tryptic and Arg-C digests of human blood plasma and tryptic digest of human protein extract show that they coincide the method optimized for trypsin[1]. The data point of the optimized method for alternative enzymes was the combined Mascot results obtained from the investigation of the Asp-N, Glu-C and chymotrypsin digests from human blood plasma results (neither the search engine nor the sample affects the results, see *Comparison of Different Samples and Search Engines*), therefore only the calculated NCE% settings of the optimized method for alternative enzymes was shown for these three enzymes.

	m/z	for 2+ peptides		for 3+ peptides	
		eV	in NCE%	eV	in NCE%
Plasma trypsin opt CE trendlines	300	13.95	26	11.65	23
	2000	84.67	24	80.50	24
Plasma Arg-C opt CE trendlines	300	14.81	27	12.25	24
	2000	87.74	24	82.63	24
Human protein trypsin opt CE trendlines	300	13.17	24	12.37	24
	2000	77.77	22	75.10	22
Optimized method for trypsin[1]	300	14.83	27	15.00	29
	2000	79.77	22	68.38	20
Optimized method for alternative enzymes	300	8.84	16	11.42	22
	2000	65.45	18	62.42	18

(1) Révész, Á.; Rokob, T. A.; Jeanne Dit Fouque, D.; Turiák, L.; Memboeuf, A.; Vékey, K.; Drahos, L. Selection of Collision Energies in Proteomics Mass Spectrometry Experiments for Best Peptide Identification: Study of Mascot Score Energy Dependence Reveals Double Optimum. J. Proteome Res.2018, 17, 1898–1906. https://doi.org/10.1021/acs.jproteome.7b00912

Table S7: Exact Numbers of Peptide Hits during Performance Tests. Performance tests were conducted with human blood plasma digested by Arg-C, chymotrypsin, Glu-C and trypsin, examined at two collision energy settings: 1) at the collision energy method previously optimized for tryptic peptides of HeLa and *E. coli*, and 2) our optimized collision energy setting for non-tryptic peptides. The latter optimized setting was collectively established for peptides produced by Asp-N, chymotrypsin and Glu-C. During the experiments, all digests were measured with 3 repetitions at both MS settings, and data were evaluated using both Byonic (upper part of the table) and Mascot (lower part of the table). For Byonic, the score limit was > 200 score and log Prob value > 2, and for Mascot > 25 score. The peptide charges were merged with Serac, not considered separately.

**Byonic (>200 score, > 2 log Prob)**	Trypsin		Arg-C		Chymotrypsin		Glu-C	
	Peptide hits	Score average	Peptide hits	Score average	Peptide hits	Score average	Peptide hits	Score average
Alt enz opt CE method rep 1	944	418.90	401	428.39	702	404.87	1022	418.31
Alt enz opt CE method rep 2	1006	434.12	405	431.43	709	397.75	1007	415.27
Alt enz opt CE method rep 3	1051	438.55	425	430.86	724	394.95	1017	408.21
Tryptic opt CE method rep 1	1044	433.30	446	442.97	619	361.97	879	391.55
Tryptic opt CE method rep 2	1073	439.15	432	444.80	629	368.63	990	384.18
Tryptic opt CE method rep 3	1074	442.89	440	451.29	634	369.98	955	386.10
Tryptic opt CE method rep 4	-	-	-	-	-	-	937	384.83
**Average for Alt enz opt CE method**	1000.33	430.53	410.33	430.23	711.67	399.19	1015.33	413.93
**Average for Tryptic opt CE method**	1063.67	438.45	439.33	446.35	627.33	366.86	940.25	386.66
**Difference between opt CE methods dividing by Tryptic opt CE method**	94.05%	98.19%	93.40%	96.39%	113.44%	108.81%	107.99%	107.05%

**Mascot (> 25 score)**	Trypsin		Arg-C		Chymotrypsin		Glu-C	
	Peptide hits	Score average	Peptide hits	Score average	Peptide hits	Score average	Peptide hits	Score average
Alt enz opt CE method rep 1	771	60.27	311	61.44	824	46.33	605	49.55
Alt enz opt CE method rep 2	834	61.09	317	63.01	842	45.51	607	49.02
Alt enz opt CE method rep 3	882	60.95	337	61.64	838	46.28	629	48.96
Tryptic opt CE method rep 1	915	60.93	374	62.74	611	44.30	469	47.01
Tryptic opt CE method rep 2	951	61.44	362	63.66	646	44.80	520	47.93
Tryptic opt CE method rep 3	967	61.57	361	64.95	643	44.79	515	48.26
Tryptic opt CE method rep 4	-	-	-	-	-	-	488	48.93
**Average for Alt enz opt CE method**	829.00	60.77	321.67	62.03	834.67	46.04	613.67	49.18
**Average for Tryptic opt CE method**	944.33	61.31	365.67	63.78	633.33	44.63	498.00	48.03
**Difference between opt CE methods dividing by Tryptic opt CE method**	87.79%	99.11%	87.97%	97.26%	131.79%	103.17%	123.23%	102.38%

Table S8: Sequence Coverages during Performance Tests. Performance tests were conducted with human blood plasma digested by Arg-C, chymotrypsin, Glu-C and trypsin, examined at two collision energy settings: 1) at the collision energy method previously optimized for tryptic peptides of HeLa and *E. coli*, and 2) our optimized collision energy setting for non-tryptic peptides. The latter optimized setting was collectively established for peptides produced by Asp-N, chymotrypsin and Glu-C. During the experiments, all digests were measured with 3 repetitions at both MS settings, and data were evaluated using Mascot (score limit > 25 score). The change in sequence coverage was examined in the case of the two methods with Serac. We investigated the proteins that were identified at least once using both of the two CE methods. The average sequence coverage values were calculated for each measurement for all these proteins. These values were then averaged over the three standard and three optimized measurements.

**Mascot (> 25 score)**	Trypsin	Arg-C	Chymotrypsin	Glu-C
Alt enz opt CE method rep 1	13.00%	10.34%	10.56%	10.12%
Alt enz opt CE method rep 2	14.19%	9.48%	10.82%	9.87%
Alt enz opt CE method rep 3	15.09%	10.15%	10.69%	9.95%
Tryptic opt CE method rep 1	15.04%	11.45%	8.65%	8.53%
Tryptic opt CE method rep 2	15.16%	10.98%	9.76%	8.54%
Tryptic opt CE method rep 3	15.12%	11.23%	9.33%	8.86%
Tryptic opt CE method rep 4	-	-	-	8.73%
**Average for Alt enz opt CE method**	14.09%	9.99%	10.69%	9.98%
**Average for Tryptic opt CE method**	15.11%	11.22%	9.25%	8.66%
**Difference between opt CE methods dividing by Tryptic opt CE method**	93.28%	89.03%	115.62%	115.18%

Higher Average Scores for Peptides with Byonic

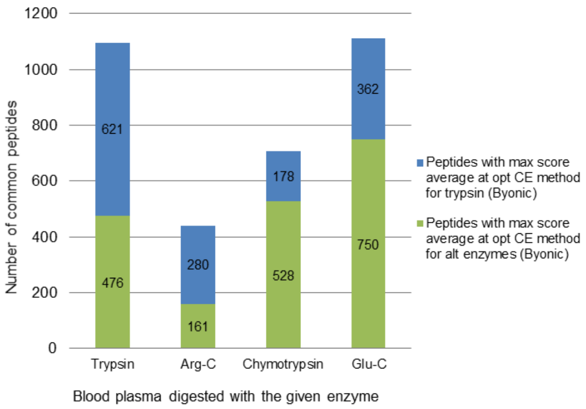

Figure S6: Number of peptides that were identified at least once by both optimized CE methods from human blood plasma for a given enzyme with Byonic. Blue bars show how many peptides have a higher average score value with the optimized CE method for trypsin, while green bars show how many peptides have a higher average score value with the CE method optimized for alternative enzymes.

Maximum Scores for Peptides with Byonic and Mascot

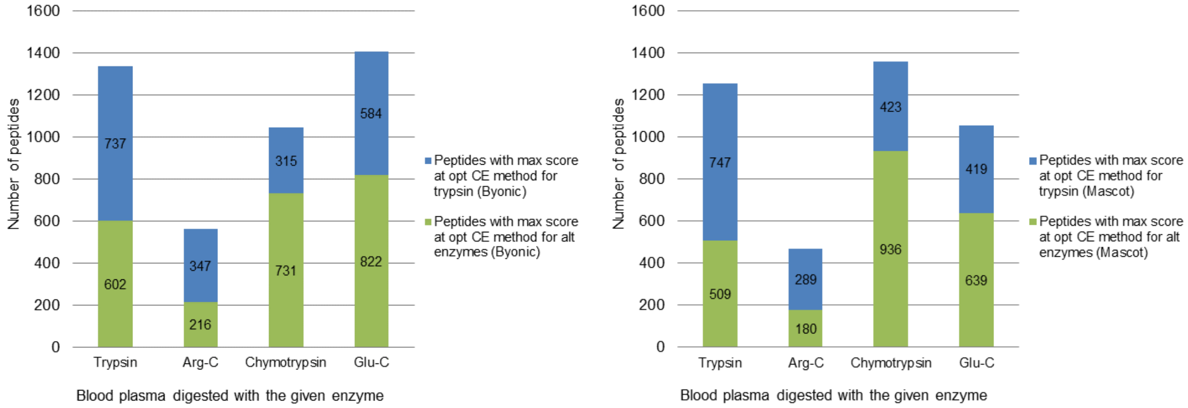

Figure S7: Number of peptides that were identified at least once by one of the optimized CE methods from human blood plasma for a given enzyme with Byonic and Mascot. Blue bars show how many peptides have the maximum score value with the optimized CE method for trypsin, while green bars show how many peptides have the maximum score value with the CE method optimized for alternative enzymes.
